# Epidemiology and Outcomes of Peptic Ulcer Perforation: An Analytical Cross-Sectional Study From a Tertiary Care Hospital in India

**DOI:** 10.7759/cureus.91206

**Published:** 2025-08-28

**Authors:** Ujala Murmu, Zenith Kerketta, Khushboo Rani, Krishna Murari, Anish Baxla, Sunny, Neyaz Ahmad, Amit Nishant, Shreya, Ajay Soreng

**Affiliations:** 1 General Surgery, Rajendra Institute of Medical Science, Ranchi, IND; 2 General Surgery, Rajendra Institute of Medical Sciences, Ranchi, IND

**Keywords:** helicobacter pylori, india, mortality, peptic ulcer perforation, socioeconomic factors, surgical outcomes, tribal health

## Abstract

Background

Peptic ulcer perforation (PUP) is a life-threatening complication of peptic ulcer disease (PUD), with significant morbidity and mortality, particularly in low-resource settings. Driven by factors such as *Helicobacter pylori* (*H. pylori*) infection, non-steroidal anti-inflammatory drugs (NSAIDs) use, and socio-economic disparities, PUP requires urgent surgical intervention. This study investigates clinical characteristics, risk factors, and surgical outcomes of PUP in a tribal predominant tertiary care hospital in India.

Methods

This analytical cross-sectional study enrolled 82 patients undergoing emergency surgery for PUP at a tertiary care centre in Ranchi, India. Patients aged >18 years with confirmed or suspected PUP were included, excluding those with sealed perforation. Data on demographics, clinical presentation, *H. pylori* status, surgical procedures, and outcomes were collected. Statistical analysis was performed using chi-square and Fisher’s exact tests, with multivariate logistic regression to identify mortality predictors.

Results

Of 82 patients, 84.14% were male, 47.56% aged 18-40 years, and 41.47% from lower socio-economic classes. Duodenal perforations (48.78%) were most common, with 82.5% testing positive for *H. pylori *(p<0.0001). Delayed presentation (>48 hours) increased mortality risk [Odds Ratio (OR)=3.50, p=0.004], with 22.44% mortality. Perforation size of >2 cm (OR=3; p=0.010)and severe anaemia (OR=2.90; p=0.013) were significant mortality predictors. Graham’s patch (n=46) had three cases of postoperative anastomotic leak, although not statistically significant (p=0.282). Complications included pleural effusion (24.39%) and surgical site infection (13.42%), with 13.42% overall mortality.

Conclusion

PUP remains a significant burden in India, driven by delayed presentation, size of perforation, and presence of severe anaemia. Early presentation of symptoms, early surgical intervention, and management of anaemia are crucial to improving outcomes.

## Introduction

Peptic ulcer disease (PUD), characterized by erosions in the gastric or duodenal mucosa that penetrate the muscularis mucosa, leading to local inflammation and potential complications, remains a significant global health challenge. Approximately 4 million individuals are diagnosed with peptic ulcers annually, with an estimated incidence of 1.5-3% worldwide. Complications such as bleeding, perforation, and obstruction occur in 10-20% of cases, with perforation affecting 2-14% of patients [1]. Peptic ulcer perforation (PUP)is a life-threatening surgical emergency, with a prevalence of approximately 5% and associated mortality rates reaching up to 30% and morbidity rates as high as 50% [2]. The condition predominantly affects the duodenum and stomach, with duodenal perforations being the most common due to high acid exposure in the first part of the duodenum [3].

The pathogenesis of PUD is multifactorial, driven by an imbalance between mucosal protective mechanisms and aggressive factors such as gastric acid hypersecretion,* Helicobacter pylori* (*H. pylori*) infection, and the use of non-steroidal anti-inflammatory drugs (NSAIDs) [4]. Additional risk factors include alcohol consumption, tobacco use, stress, and, less commonly, conditions like Zollinger-Ellison syndrome or gastric neoplasms [4]. *H. pylori *infection, in particular, is strongly associated with PUP, especially in regions with low socioeconomic status, where poor hygiene and overcrowding exacerbate transmission [5]. 30 to 50% of Gastro-duodenal perforations in the elderly with comorbidities are due to NSAID use [6].

Despite advancements in medical management, including proton pump inhibitors and *H. pylori* eradication therapy, PUP remains a critical concern due to its acute presentation and high complication rates. The classical clinical triad of sudden onset abdominal pain, tachycardia, and abdominal rigidity often signals perforation, necessitating urgent surgical intervention [7]. The recurrence rate of perforation, reported at 12.2% in long-term follow-up studies, underscores the persistent challenge of managing underlying risk factors [8]. Moreover, delayed presentation beyond 24 hours of perforation larger than 1 cm significantly increases morbidity and mortality [9].

Epidemiologically, PUP is more prevalent in males and peaks in the fifth decade of life, potentially due to lifestyle factors such as smoking and alcohol use, as well as occupational stress [10]. Jharkhand, being the tribal predominant state with economic disparities further exacerbating the burden, with higher incidence rates in the lower income population linked to limited healthcare access and higher *H. pylori* prevalence [5]. Given the significant morbidity and mortality associated with PUP, there is an urgent need to understand its contemporary patterns, risk factors, and outcomes to inform clinical practice and public health strategies.

This study aims to investigate the clinical characteristics, risk factors, and surgical outcomes of peptic ulcer perforation in a tertiary care hospital of a tribal predominant area of Jharkhand, with an emphasis on identifying factors influencing patient prognosis. Objectives of the study were: to evaluate the clinical characteristics and risk factors associated with peptic ulcer perforation; to assess the surgical outcomes and postoperative complications of PUP; to investigate the association between *H. pylori* infection and site of peptic ulcer perforation.

## Materials and methods

Study design and setting

This analytical cross-sectional study was conducted from March 2024 to February 2025 in the department of general surgery of a tertiary care hospital in Ranchi, India. Patients undergoing emergency operations for PUP were the population under study.

Sampling and sample size

A purposive sampling approach was used to enrol participants with confirmed peptic ulcer perforation, based on clinical and diagnostic criteria (e.g., endoscopic or surgical confirmation), to ensure the study population was representative of the target group. The sample size was calculated using the prevalence of peptic ulcer perforation (5%) reported by Chung et al. [3]. For a single proportion, the sample size of 76 was determined using the formula n=Z^2^p(1-p)/E^2^, where Z=1.96 (for a 95% confidence level), p=0.05 (prevalence), and E=0.05 (margin of error). Accounting for a 10% non-response rate, based on similar studies, the final sample size was adjusted to 82 participants.

Eligibility criteria

The participants above 18 years with PUP or suspected cases of PUP who underwent emergency operation with patients presented with acute abdomen along with features of peritonitis confirmed either by X-ray abdomen erect or CT scan of abdomen, were included. Severely ill PUP patients with sealed peptic ulcer perforation were excluded from the study.

Method of sample collection

The participant or a legally authorised representative (in case of the participant's inability to give consent) had to fill out a written informed consent. Detailed history, clinical examination, baseline, and specific investigations for pre-anaesthesia assessment (complete blood count, x-ray abdomen erect and supine, arterial blood gas analysis, serology) were recorded by the staff.

Complete details of the clinical procedure, sample processing (as and where applicable), namely, intra-operative findings, were noted with special reference to the site of perforation. A blood sample was collected, and a rapid *H. pylori* antibody test was done. Clinical judgments of recovery status were made by early ambulation, early normalization of bowel and bladder habits, and any evidence of surgical site infection, obstruction. 

Data collection and statistical analysis

A standard template was made in Microsoft Excel 2023 to enter the data collected in the research. *Jamovi* software version 2.6.23 (The jamovi Project, Sydney, Australia) was used for further analysis. The qualitative data were expressed in numbers and proportions. Chi-square tests of association with Fisher's exact test correction were applied to explore association, and multivariate logistic regression was performed to assess independent predictors of mortality based on previous literature [2,5,9].

Ethical Consideration: The study was ethically cleared by the Institutional Ethics Committee, Rajendra Institute of Medical Sciences, Ranchi (Memo no. 285/IEC, RIMS Dated: 20/03/2024).

## Results

Table 1 presents demographic profiles, clinical presentations, and risk factors of 82 patients with PUP. The majority were male (69;84.14%), aged 18-40 years (39;47.56%), and from lower socioeconomic classes (34;41.47%). Farmers (33;40.24%) and alcohol users (35;42.68%) were prevalent. Abdominal pain (76;92.68%) and peritonitis (75;91.46%) were the most common presenting complaints and signs, respectively. Severe anemia (23;28.05%) and gas under the diaphragm (80;97.56%) were frequent findings, with the first part of the duodenum being the most common site of perforation (40;48.78%). *H. pylori* positivity was observed in (45;54.88%) of patients.

**Table 1:** Demographic and clinical characteristics of patients with PUP *According to the modified BG Prasad classification of socio-economic status for the year 2024. PUP: peptic ulcer perforation.

Variable	Category	Number	Frequency (%)
Sex	Male	69	84.14
	Female	13	15.86
Age Group (Years)	18–40	39	47.56
	41–60	32	39.02
	>60	11	13.42
Socioeconomic Class	Upper	1	1.22
	Upper Middle	7	8.53
	Lower Middle	16	19.52
	Upper Lower	24	29.26
	Lower	34	41.47
Occupation	Government Employee	3	3.66
	Self-Employed	22	26.83
	Unemployed	11	13.42
	Farmer	33	40.24
	Student	13	15.85
Addiction	Tobacco Smoking	20	24.40
	Alcohol	35	42.68
	Tobacco Chewing	30	36.58
Risk Factor	Chronic NSAID Intake	17	20.73
Presenting Complaints	Abdominal Pain	76	92.68
	Vomiting	1	1.21
	Abdominal Distension	10	12.19
	Others	33	40.24
Signs	Peritonitis	75	91.46
	Shock	23	28.04
	Fever	8	9.75
	SpO2 <90%	24	29.26
Level of Hemoglobin (WHO Classification of Anaemia)	Normal (Male >/=13 g/dl; Female >/=12g/dl)	37	45.13
	Mild Anaemia (Male 11-12.9 g/dl; Female 11-11.9 g/dl)	13	15.85
	Moderate Anaemia (both Male and Female 8-10.9 g/dl)	9	10.97
	Severe Anaemia (both Male and Female <8g/dl)	23	28.05
WBC Count (/cumm)	<4500	0	0.00
	4500–11000	69	84.15
	>11000	13	15.85
Gas Under Diaphragm	Yes	80	97.56
	No	2	2.44
Site of Perforation	Body of Stomach	0	0.00
	Antral	19	23.18
	Pyloric	23	28.04
	1st Part of Duodenum	40	48.78
Size of Perforation	<1.0 cm	31	37.80
	1.0–2.0 cm	46	56.10
	>2.0 cm	5	6.10
Rapid *H. Pylori* Antibody Test	Negative	37	45.12
	Positive	45	54.88
Type of Surgery	Gastrojejunostomy	1	1.22
	Modified Graham’s Patch	34	41.46
	Graham’s Patch	47	57.32

Table 2 illustrates the relationship between *H. pylori* infection and perforation site. Of 40 duodenal perforations, 33 tested positive for *H. pylori* antibodies compared to 12 of 42 stomach perforations. The association was statistically significant (p=0.0001), indicating a stronger link between. *H. pylori* positivity and duodenal perforations.

**Table 2:** Association of H. pylori antibody test with site of PUP Chi-square test of association is performed. PUP: peptic ulcer perforation.

	Duodenal	Gastric (Antrum + pylorus)	Test staistic (df)	p-value
Positive	33	12	24.06 (1)	<0.0001
Negative	7	30		

Table 3 examines the relationship between surgical procedures and postoperative leak. Among 46 patients undergoing Graham’s patch, three experienced post-operative anastomotic leak; however, no leak occurred in one gastrojejunostomy or modified Graham’s patch cases, although this association is not significant (p=0.282).

**Table 3:** Association of operative procedure with postoperative leak * Chi-square test with Fischer's exact correction was applied to obtain result.

Operative Procedure	Leak (Yes)	Leak (No)	Statistic (df)	p-value*
Graham’s Patch	3	43	2.437 (2)	0.282
Modified Graham’s Patch	0	35		
Gastrojejunostomy	0	1		

Table 4 analyses the impact of symptom onset duration on mortality. Of 45 patients with symptoms >48 hours, 11 died compared to 1 of 12 with symptoms <24 hours, and none of 25 with symptoms 24-48 hours. The association was statistically significant (p=0.010), suggesting delayed presentation increases mortality risk.

**Table 4:** Association of duration of symptom onset with mortality * Chi-square test of association with Fisher's exact correction.

Duration of Symptom Onset	Discharged	Death	Statistic (df)	p-value
<24 hours	11	1	8.134 (2)	0.010*
24–48 hours	25	0		
>48 hours	34	11		

The multivariate logistic regression model in Table 5 assessed predictors of mortality (Death vs. Discharge) in patients. The model included symptom duration, severe anemia, shock, age group, and *H. pylori *perforation size as predictors. The model was significant (chi-square=15.32, p= 0.009, Nagelkerke R^2^= 0.24). The analysis showed patients with symptoms >48 hours had 3.50 times higher odds of mortality compared to <24 hours (p=0.004), confirming delayed presentation as a significant risk factor. Severe anaemia increased mortality odds by 2.90 times (p=0.013), indicating its role in poor prognosis. Patients with shock had 2.20 times higher odds of mortality (p=0.067). Older age showed elevated odds [odds ratio (OR)=2.10] but not significant (p=0.108). *H. pylori* has no significant effect on mortality (p=0.735)while Perforation of >2cm showed significantly high odds of mortality (OR=3; p=0.010).

**Table 5:** Independent predictors of mortality in patients of peptic ulcer perforation OR: odds ratio, CI: confidence interval.

Predictor	OR	95% CI	p-value
Symptom Duration 24–48hrs	0.85	0.10–7.20	0.881
Symptom Duration >48hrs	3.50	1.50–8.15	0.004
Severe Anaemia Yes	2.90	1.25–6.75	0.013
Shock Yes	2.20	0.95–5.10	0.067
Age Group 41–60	1.40	0.60–3.25	0.435
Age Group >60	2.10	0.85–5.20	0.108
H pylori Positive	1.15	0.50–2.65	0.735
Perforation Size >2cm	3.00	1.30–6.90	0.010

Table 6 summarizes the outcomes and complications. Graham’s patch was the most common procedure (47;57.32%), followed by Modified Graham’s Patch (34;41.46%). Postoperative complications included pleural effusion (20;24.39%), surgical site infection (11;13.42%), and leak (3;3.65%). Most patients had a hospital stay of seven to 14 days, with (71;86.58%) discharged on improvement and (11;13.42%) mortality.

**Table 6:** Outcomes and complications

Category	Subcategory	Number	Frequency (%)
Outcome	Death	11	13.42
	Discharged on Improvement	71	86.58
Hospital Stay Duration	>14 days	4	4.88
	7–14 days	64	78.04
	<7 days	14	17.08
Post-operative Complications	Surgical Site Infection	11	13.42
	Pleural Effusion	20	24.39
	Leak	3	3.65

## Discussion

The study provides extensive information regarding the epidemiology, clinical presentation, and surgical outcome of peptic ulcer perforation (PUP) in a tertiary care facility of Ranchi, India. Results reflect trends in the rest of the world, coupled with issues that can be seen as region-based, especially the prevalence of *H. pylori* infection in this area, as well as socioeconomic forces behind late onset and death.

Demographic characteristic of our study portrays a predominance of males (69;84.14%), with ages 18-40 years (39;47.56%) is also in line with the previous research findings [9]. The high prevalence of smoking and alcohol use can be attributed to the number of PUP cases being more dominant in males in our study (35;42.68%). Farmers (33;40.24%) formed a substantial percentage, and probably due to occupational stress and lack of access to services in rural locations, the study results were supported by Levenstein et al. [10].

Duodenal perforations were highly prevalent among patients infected with *H. pylori* (p<0.0001), with (33;82.5%) of the duodenal perforation patients testing positive for *H. pylori* antibodies, as opposed to (12;28.5%) of stomach patients. The overall prevalence of PUP in patients with peptic ulcer is 5% in the previous study from Singapore [3]. However, the antibody test is not a diagnostic test for *H. pylori* infection; this method was used due to its feasibility. This is in line with data showing that *H. pylori* is one of the leading causes of duodenal ulcers as it enhances the secretion of gastric acid and weakens the mucosal barriers [4]. This is further supported by a study which reported 70-90 % prevalence of* H. pylori* in cases of duodenal ulcer in low-income settings [11]. The lower association with gastric perforations in our study may reflect the increasing role of NSAIDs, which are implicated in 30-50% of gastric ulcers, particularly in elderly populations [6].

Delayed presentation >48 hours was a significant predictor of mortality (OR=3.50, p=0.004), a finding consistent with [2] Buck et al., who identified surgical delay as a critical determinant of survival. A 2021 study in South Asia further emphasized that delayed presentation, often due to poor healthcare access and low health literacy, exacerbates peritonitis and septic shock, increasing mortality [12]. Severe anaemia and shock were additional independent predictors of mortality, underscoring the need for early recognition and management of these complications.

In tribal settings like Jharkhand, barriers to early presentation include low health literacy, which delays recognition of PUP symptoms, and limited transport infrastructure, hindering timely access to healthcare [13]. These factors exacerbate delayed presentation, underscoring the need for community-based education and improved rural transport to enhance early intervention. Perforation of >2 cm was found to be a significant predictor of mortality as (OR=3, p=0.010) suggested by the study done by Bodepudi et al. [14].

Surgical outcomes revealed Graham’s patch as the most common procedure (n=46), with 3 post-operative anastomotic leaks reported, while modified Graham’s patch and gastrojejunostomy showed none, though the difference was not statistically significant (p=0.282). These findings support the efficacy of omental patch repairs, as reported by Chung and Shelat 2017, who noted their simplicity and effectiveness in emergency settings [3]. The absence of leaks in gastrojejunostomy cases (p=0.013) may reflect its selective use in complex cases, though the small sample size limits its generalizability.

Postoperative complications included pleural effusion (20;24.39%) and surgical site infection (11;13.42%), comparable to global rates [9]. The 13.42% mortality rate, lower than the global 20-30 [3], reflects improved care but highlights access issues. Socioeconomic disparities, with (34;41.47%) from lower classes, exacerbate the PUP burden [1,15].

Limitations include the cross-sectional design precluding the establishment of causality and reliance on rapid *H. pylori* tests, which may not confirm active infection. Additionally, no inflammatory markers were assessed in the study, which could limit its interpretation. This was a single-center study with a comparatively small sample size, which may limit its generalizability. Future studies should use more accurate diagnostics and multi-center data.

## Conclusions

This study confirms that peptic ulcer perforation (PUP) poses a significant burden in a tertiary care setting in India, primarily affecting young males from lower socioeconomic groups, with duodenal perforation's predominance. Delayed presentation (>48 hours), size of perforation, and severe anaemia were key mortality predictors. Modified Graham’s patch and Gastrojejunostomy were effective, with low leak rates, but it's generalizability is restricted due to limited sample size for each procedure. High *H. pylori* positivity was noted, particularly in duodenal perforations, but was not significantly associated with mortality. These findings underscore the need for early surgical intervention and management of anaemia and shock to improve outcomes in resource-limited settings.

## References

[1] Abbasi-Kangevari M, Ahmadi N, Fattahi N (2022). Quality of care of peptic ulcer disease worldwide: a systematic analysis for the global burden of disease study 1990-2019. PLoS One.

[2] Buck DL, Vester-Andersen M, Møller MH (2013). Surgical delay is a critical determinant of survival in perforated peptic ulcer. Br J Surg.

[3] Chung KT, Shelat VG (2017). Perforated peptic ulcer - an update. World J Gastrointest Surg.

[4] Hooi JK, Lai WY, Ng WK (2017). Global prevalence of helicobacter pylori infection: systematic review and meta-analysis. Gastroenterology.

[5] Lanas A, Chan FK (2017). Peptic ulcer disease. Lancet.

[6] Lau JY, Sung J, Hill C, Henderson C, Howden CW, Metz DC (2011). Systematic review of the epidemiology of complicated peptic ulcer disease: incidence, recurrence, risk factors and mortality. Digestion.

[7] Malfertheiner P, Chan FK, McColl KEL (2009). Peptic ulcer disease. Lancet.

[8] Møller MH, Adamsen S, Thomsen RW, Møller AM (2011). Multicentre trial of a perioperative protocol to reduce mortality in patients with peptic ulcer perforation. Br J Surg.

[9] Søreide K, Thorsen K, Harrison EM, Bingener J, Møller MH, Ohene-Yeboah M, Søreide JA (2015). Perforated peptic ulcer. Lancet.

[10] Levenstein S, Ackerman S, Kiecolt-Glaser JK, Dubois A (1999). Stress and peptic ulcer disease. JAMA.

[11] Malaty HM, El-Kasabany A, Graham DY (2002). Age at acquisition of Helicobacter pylori infection: a follow-up study from infancy to adulthood. Lancet.

[12] Siri KN, Arpita BS, Neelendra MVN (2021). Burden of peptic ulcer disease among patients with upper gastrointestinal bleeding. Int J Surg Sci.

[13] Deb Roy A, Das D, Mondal H (2023). The tribal health system in India: challenges in healthcare delivery in comparison to the global healthcare systems. Cureus.

[14] Bodepudi AK, Voruganti MR, Kanuru C, Sangani V (2022). Study of factors to assess the mortality and morbidity in perforated peptic ulcer disease. Int J Surg Sci.

[15] Xie X, Ren K, Zhou Z, Dang C, Zhang H (2022). The global, regional and national burden of peptic ulcer disease from 1990 to 2019: a population-based study. BMC Gastroenterol.

